# Mechanical thrombectomy for acute ischemic stroke: systematic review and meta-analysis

**DOI:** 10.31744/einstein_journal/2022RW6642

**Published:** 2022-08-02

**Authors:** Ananda Jessyla Felix Oliveira, Sônia Maria Nunes Viana, André Soares Santos

**Affiliations:** 1 Escola de Enfermagem Universidade Federal de Minas Gerais Belo Horizonte MG Brazil Escola de Enfermagem , Universidade Federal de Minas Gerais , Belo Horizonte , MG , Brazil .; 2 Faculdade de Ciências Econômicas Universidade Federal de Minas Gerais Belo Horizonte MG Brazil Faculdade de Ciências Econômicas , Universidade Federal de Minas Gerais , Belo Horizonte , MG , Brazil .

**Keywords:** Thrombolytic therapy, Mechanical thrombolysis, Thrombectomy, Ischemic stroke, Systematic review

## Abstract

**Objective:**

To evaluate the safety and efficacy of mechanical thrombectomy associated with standard medical treatment compared with standard medical treatment only to treat patients with acute ischemic stroke.

**Methods:**

This was a systematic review and metaanalysis of randomized controlled trials. An electronic search was performed in the following databases: MEDLINE ^®^ /PubMed ^®^ , Cochrane Library (Trials), LILACS/IBECS (via *Biblioteca Virtual em Saúde* (BVS)) and Embase. Complementary searches were also conducted. The selection of studies and data collection were done by two investigators independently.

**Results:**

The final analysis included 16 publications related to 15 studies. The mechanical thrombectomy was associated to a reduction in the risk of death of all cause (16.81% *versus* 20.13%; relative risk of 0.85; p=0.04), improvement in the number of patients with functional independence after 90 days (45.65% *versus* 27.45%; relative risk of 1.65; p<0.01), and improvement in the rate of revascularization (76.2% *versus* 33.85%; relative risk of 2.20; p<0.01). There was no significant difference in terms of symptomatic intracranial hemorrhage (4.78% *versus* 3.88%; relative risk of 1.27; p=0.21).

**Conclusion:**

Mechanical thrombectomy associated with standard medical treatment seem to be safe and effective to treat patients with acute ischemic stroke compared with standard medical treatment only.

## INTRODUCTION

Ischemic stroke (IS) is characterized by disruption of blood supplying to the brain, retina or spinal cord, usually caused by an embolus or thrombus. ^( 1 - 5 )^ In the area supplied by the obstructed vessel there is a reduction of blood flow that leads to injury of adjacent tissues. This ischemic area may consist of dead tissue which cannot be recovered (ischemic core) or by the affected tissue that is still recoverable (penumbra) if an immediate reperfusion is performed. ^( 1 - 3 , 5 , 6 )^ The IS is considered the most common type of stroke. ^( 7 , 8 )^

The 2016 Global Burden of Disease (GBD) contributors pointed out that the overall lifetime risk of stroke starting at age of 25 years is approximately 25%. ^( 9 )^ Between the years 1990 and 2016, there was a reduction in the mortality rate and overall incidence of stroke, however, the burden of the disease remained high. In 2016, a total of 13.7 million new cases worldwide (95% confidence interval - 95%CI: 12.7-14.7) appeared, 70% of which were ischemic strokes. ^( 10 )^ In that same year, strokes were the second leading cause of death with 5.5 million cases (95%CI: 5.3-5.7). Of these, 2.7 million were ischemic (95%CI: 2.6-2.8). ^( 10 )^ In Brazil, in 2016, stroke was responsible for 61.8% (95%CI: 61.5-62.1%) of deaths due to stroke and 814.66 disease-adjusted life years (DALYs), *i.e* . years of healthy life lost to disorde per 100,000 population among men and 490.28 among women. ^( 11 )^

Early diagnosis and timely treatment can reduce stroke sequelae. ^( 5 , 6 )^ One of the main therapeutic options for acute stroke is the standard medical treatment used to treat acute ischemic stroke. This treatment includes ventilatory support, supplemental oxygen, temperature control, blood glucose and blood pressure control, antiplatelet agents, anticoagulants, and intravenous thrombolysis (IT) using recombinant tissue plasminogen activator (rt-PA). ^( 12 - 16 )^

The other treatment possibility is the association of standard medical treatment with mechanical thrombectomy (MT). ^( 5 , 17 , 18 )^ Mechanical thrombectomy is based on the insertion of an endovascular catheter and other devices for the extraction or fragmentation of the thrombus that is occluding an intracranial artery. This procedure is often conducted by puncturing of the femoral artery with the patient under general anesthesia or sedation. ^( 5 , 17 )^

The treatment, hospitalization, and rehabilitation of patients with IS generate high costs for health care systems. ^( 19 - 21 )^ In a study evaluating the costs during hospitalization for IS in the United States, the mean total costs were US$ 68,370 for patients who died at discharge, US$ 73,903 for patients discharged with disability, and US$ 24,448 for patients discharged without disability (p<0.001). ^( 22 )^

Mechanical thrombectomy has a high cost when compared with IT. ^( 19 )^ A prospective study in private hospitals in the city of Joinville, Santa Catarina, Brazil, showed that the mean cost for patients with IS who received IT was US$ 11,463 (interquartile range - IQR of 8,931-14,291) and for patients who received IT and underwent MT the cost was US$ 37,948 (IQR of 32,697-47,205). ^( 23 )^ However, better outcomes have also been attributed to MT. ^( 19 , 24 , 25 )^ In this scenario, robust studies evaluating the efficacy and safety of treatments are necessary to support decisions regarding the treatment of IS, as well as to evaluate important short- and long-term outcomes.

## OBJECTIVE

To evaluate the safety and efficacy of mechanical thrombectomy associated with standard medical treatment for the treatment of patients with acute ischemic stroke compared with standard medical treatment only.

## METHODS

This report followed the principles of the Preferred Reporting Items for Systematic Reviews and Meta-Analysis (PRISMA) consensus. ^( 26 - 28 )^

### Research question

Is MT associated with standard medical treatment safe and effective for the treatment of patients with acute ischemic stroke when compared with the use of standard medical treatment only? The question in PICO (Population, Intervention, Comparison, Outcome) format is available in appendix A.

### Literature search

A systematic search using various keywords was conducted in the MEDLINE ^®^ /PubMed ^®^ , Cochrane Library (Trials), *Literatura Latino-Americana e do Caribe em Ciências da Saúde* (LILACS) and *Índice Bibliográfico Español en Ciencias de la Salud* (IBECS) via *Biblioteca Virtual em Saúde* (BVS) and Embase. The complete search strategies are available in appendix B. A complementary search was also conducted on ClinicalTrials.gov, Google Scholar, and conference abstracts of the area. References were imported into Rayyan QCRI (rayyan.qcri.org) ^( 29 )^ to select papers and remove duplications.

### Data collection and selection of studies

Data collection and selection of studies were done by two independent researchers in the three phases. Disagreements in each phase were resolved during consensus. In phase 1, references were evaluated for title and abstract. In phase 2, the full texts of the references that were selected in phase 1 were retrieved and evaluated in their entirety for inclusion. In phase 3, data collection was performed for the outcomes of interest in the articles selected in phase 2. ^( 30 )^

### Inclusion and exclusion criteria

Randomized clinical trials that compared MT associated with standard medical treatment and the use of standard medical treatment only in patients who suffered acute ischemic stroke were included. Studies that MT was performed with the help of intra-arterial thrombolysis in more than 60% of the participants and who received treatment in the intervention group were excluded. This criterion was used so that the effect of this treatment would not interfere in the measurement of the efficacy and safety of MT. Studies that used, in most participants, first-generation devices that was considered inferior than second-generation devices were also excluded. ^( 31 , 32 )^ No restrictions were imposed in terms of date, language, or local of the study.

### Evaluated outcomes

The outcomes evaluated were functional independence after 90 days of treatment with Modified Rankin Scale (mRS) score from zero to two (mRS 0-2); revascularization rate; all-cause mortality, and symptomatic intracranial hemorrhage. The mRS measures disability in stroke patients ranged from zero (no symptoms) to six (death). ^( 33 )^

### Data analysis

Data collection was conducted using an electronic spreadsheet. A qualitative synthesis of the results was performed by aggregating data from different studies. A quantitative synthesis of the clinical outcomes was performed using the R software, ^( 34 )^ the inverse variance method, and a random effects model by means of the method of DerSimonian et al. ^( 30 , 35 , 36 )^ Analyses were performed using the ‘meta’ package. ^( 37 )^ The dichotomous outcomes were presented by means of the relative risk analysis (RR), with the 95%CI as measures of association. Results with p value <0.05 were considered statistically significant. Analyses with I ^2^ >30% presented moderate heterogeneity, I ^2^ >50% presented substantial heterogeneity, and I ^2^ >75% presented high heterogeneity. Heterogeneity data with a p value of the χ ^2^ test <0.10 were considered statistically significant. ^( 30 )^

### Assessment of methodological quality of included studies

To assess the quality of methods of the studies, the RoB 2.0 tool (revised Cochrane risk-of-bias tool for randomized trials) was used. ^( 38 )^ This tool is composed of five domains that assess in randomized clinical trials the following: biases arising from the randomization process, deviations of the intervention of interest, presence of incomplete data, measurement of outcomes, and reporting of results. All domains are required, and none should be added. After answering the guiding questions, the domains can be evaluated in three categories: low risk of bias (LRoB), some concerns (SC) or high risk of bias (HRoB). The tool does not provide a score. ^( 30 , 38 )^ The risk of bias assessment of the primary studies was done in duplicate, and divergent results were reevaluated until a consensus decision was reached. The Grading of Recommendations Assessment, Development and Evaluation (GRADE) system was used to assess the level of evidence. ^( 30 , 39 - 44 )^ In general, prospective studies, with contemporary control, randomized, with larger number of participants and masking, generate higher levels of evidence. ^( 45 )^ Publication bias was assessed by visual inspection of funnel plots and Egger’s test when, in the meta-analysis, there was at least 10 studies with data on the studied outcome. ^( 30 , 36 )^

## RESULTS

### Selection of studies

**Figure 1:**
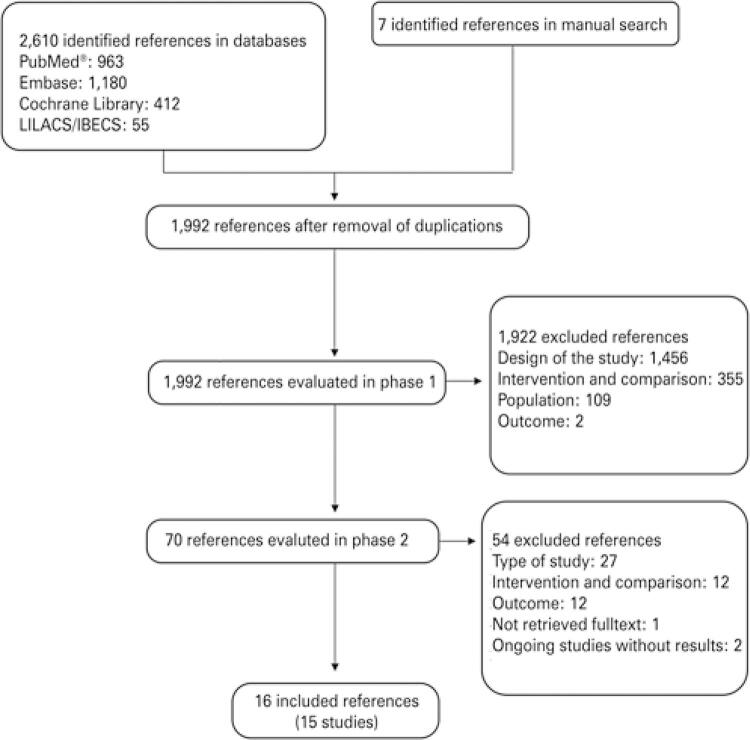
Flowchart for selection of studies

LILACS: *Literatura Latino-Americana e do Caribe em Ciências da Saúde; IBECS: Índice Bibliográfico Español en Ciencias de la Salud.*

A total of 2,617 references were identified in the search. A total of 70 references were evaluated, 54 of which were excluded. Among the excluded references, 21 were from 14 studies. In addition, two references were excluded because the study was still in progress and had no results. Another study was excluded because the full text was not retrieved and the abstract contained no results (Appendixes C to E). For the final analysis and data collection, 16 publications related to 15 studies were included ( Figure 1 ).

### Characteristics of included studies

Of the 15 studies included, five evaluated only patients eligible for IT with alteplase, ^( 46 - 50 )^ 14 studies mainly evaluated occlusion of large vessels, and one study evaluated vertebrobasilar artery occlusion. ^( 51 )^ In 6 studies, the retriever stent was used in all patients treated in the intervention group. ^( 46 , 48 , 52 - 56 )^ The studies were conducted in 16 countries: Australia, Austria, Brazil, Canada, China, Denmark, France, Germany, Ireland, Netherlands, New Zealand, South Korea, Spain, Switzerland, the United Kingdom, and the United States. Twelve studies were multicenter and three were single-center. ^( 53 , 56 , 57 )^ One study was conducted in Brazil. ^( 58 )^ Most studies evaluated relatively small samples (100 patients or less: five studies; 100 to 200 patients: four studies; 200 to 300 patients: three studies; more than 300: three studies). Nine studies were finished early: eight due to efficacy of the intervention in other studies ^( 46 - 50 , 53 - 55 , 59 )^ and one for excessive crossover. ^( 51 )^ The study with the most randomized patients was MR CLEAN (500 patients). ^( 60 )^ One study was available only as a conference abstract. ^( 56 )^ General characteristics of the included studies are available in appendices F and G.

### Outcome analysis

#### All-cause mortality

**Figure 2:**
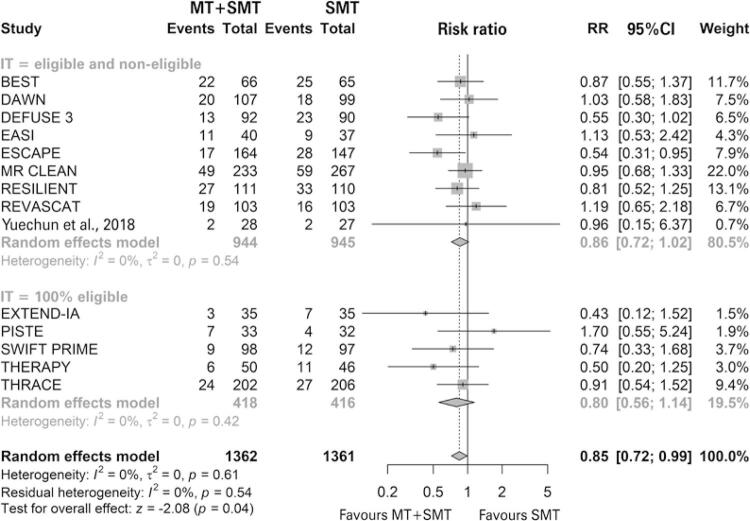
Forest plot of the meta-analysis of the all-cause mortality outcome, stratifying studies by eligibility criteria for intravenous thrombolysis

MT: mechanical thrombectomy; SMT: standard medical treatment; RR: relative risk; 95% CI: 95% confidence interval; IT: intravenous thrombolysis.

A total of 14 studies were included in the mortality analysis, five of which had a population that was 100% eligible for IT. ^( 46 - 50 )^ Ten studies presented data that tended to favor MT associated with standard medical treatment, but only one reached statistical significance. ^( 59 )^ Only one study reported mortality data within 30 days. ^( 56 )^ The other 13 reported data within 90 days. In the meta-analysis, MT associated with standard medical treatment significantly reduced the risk of patient death compared with standard medical treatment alone (16.81% *versus* 20.13%; RR of 0.85; 95%CI: 0.72-0.99; p=0.04; I ^2^ =0%, p=0.61; 14 studies, 2,723 patients) ( Figure 2 ).

**Figure 3:**
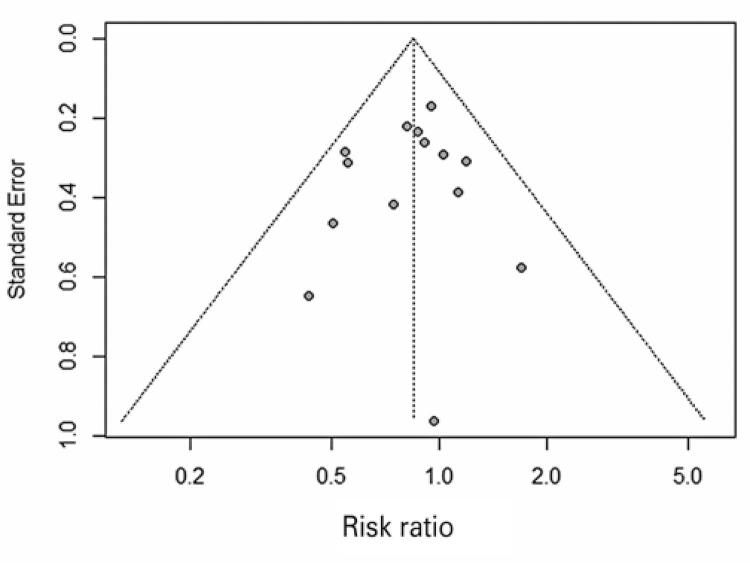
Funnel plot of outcome all-cause mortality

In the subgroup analysis, patients were stratified regarding eligibility for IT. In this analysis, we observed that there was no difference between the intervention and the comparator in the “eligible and non-eligible” subgroup (19.07% *versus* 22.54%; RR of 0.86; 95%CI: 0.72-1.02; I ^2^ =0%; p=0.54; nine studies; 1,889 patients), as well as in the “100% eligible” subgroup (11.72% *versus* 14.66%; RR of 0.80; 95%CI: 0.56-1.14; I ^2^ =0.42%; p=0.42; five studies; 834 patients). Furthermore, no difference was observed between subgroups as the CIs intersect. Heterogeneity was null. No statistically significant publication bias was observed (p=0.6137) ( Figure 3 ).

#### Functional independence after 90 days of treament

**Figure 4:**
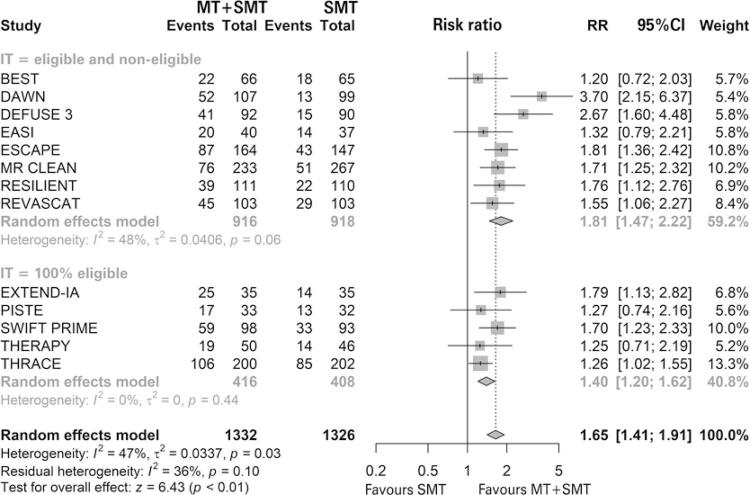
Forest plot of the meta-analysis of the functional independence after 90 days of treatment with Modified Rankin Scale (mRS), stratifying studies by eligibility criteria for intravenous thrombolysis

MT: mechanical thrombectomy; SMT: standard medical treatment; RR: relative risk; 95%CI: 95% confidence interval; IT: intravenous thrombolysis.

Thirteen studies were included in the functional independence analysis, of which five had a population 100% eligible for IT. ^( 46 - 50 )^ All studies evaluated for this outcome showed data within 90 days that tended to favor MT, and four studies did not reach statistical significance. ^( 47 , 49 , 51 , 53 )^ In the meta-analysis, MT associated with standard medical treatment significantly increased the risk of patients being independent compared with standard medical treatment only (45.65% *versus* 27.45%; RR of 1.65; 95%CI: 1.41-1.91; p<0.01; I ^2^ =47%; p=0.03; 14 studies; 2,658 patients) ( Figure 4 ).

**Figure 5:**
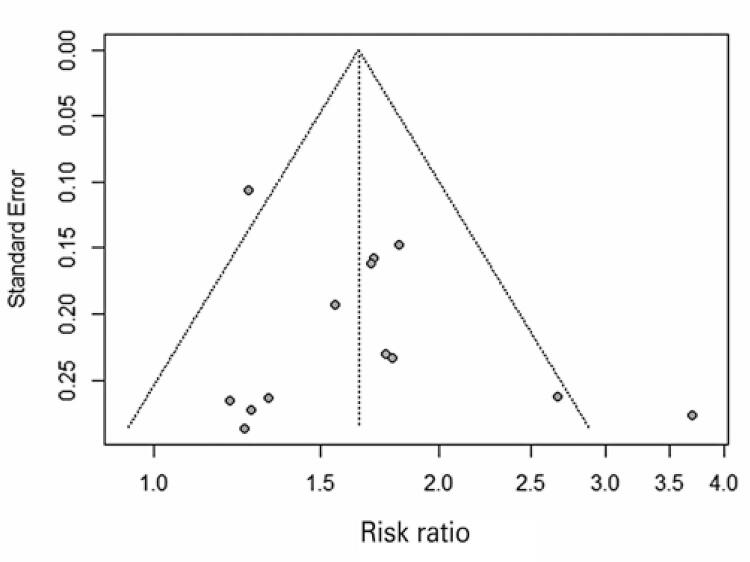
Funnel plot of outcome functional independence after 90 days of treatment with Modified Rankin Scale (mRS)

However, heterogeneity was moderate and significant. In the subgroup analysis, patients were stratified as to eligibility for IT. There was a difference between the intervention and the comparison in the eligible and non-eligible subgroup (41.70% *versus* 22.33%; RR of 1.81; 95%CI: 1.47-2.22; I2=48%; p=0.06; 8 studies; 1,834 patients) and in the “100% eligible” subgroup (54.33% *versus* 38.97%; RR of 1.40; 95%CI: 1.20-1.62; I2=0%; p=0.44; 5 studies; 824 patients). Furthermore, no difference was observed between subgroups. No statistically significant publication bias was observed (p=0.2339) ( Figure 5 ).

#### Symptomatic intracranial hemorrhage

**Figure 6:**
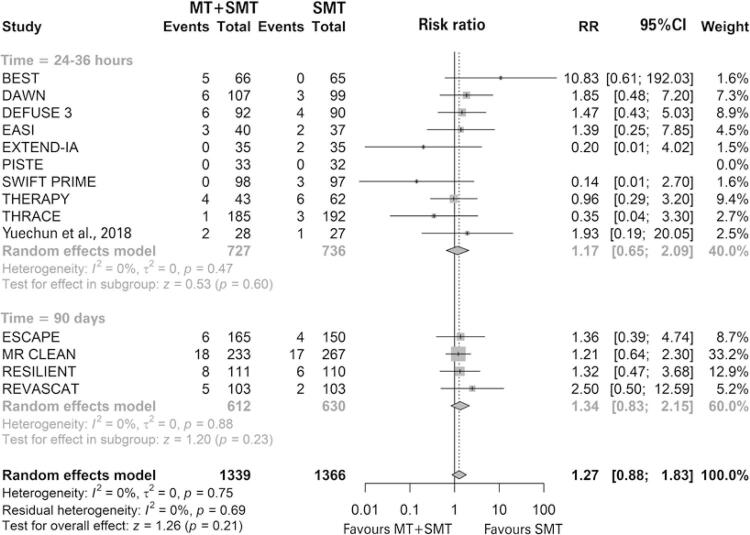
Forest plot of the meta-analysis of the outcome symptomatic intracranial hemorrhage, stratifying studies by time of assessment of the outcome

MT: mechanical thrombectomy; SMT: standard medical treatment; RR: relative risk; 95% CI: 95% confidence interval.

Fourteen studies were included in the analysis of symptomatic intracranial hemorrhage, of these, 10 studies were evaluated between 24 and 36 hours and the other four at 90 days. ^( 54 , 58 - 60 )^ In one study, no events were observed for this outcome. ^( 47 )^ In none of the other 13 studies was statistical significance demonstrated, but four studies tended to favor MT associated with standard medical treatment. ^( 46 , 48 - 50 )^ In the meta-analysis, no significant difference was observed between intervention and comparasion regarding the risk of patients presenting the outcome (4.78% *versus* 3.88%; RR of 1.27; 95%CI: 0.88-1.83; p=0.21; I ^2^ =0%; p=0.75; 14 studies; 2,705 patients) ( Figure 6 ).

**Figure 7:**
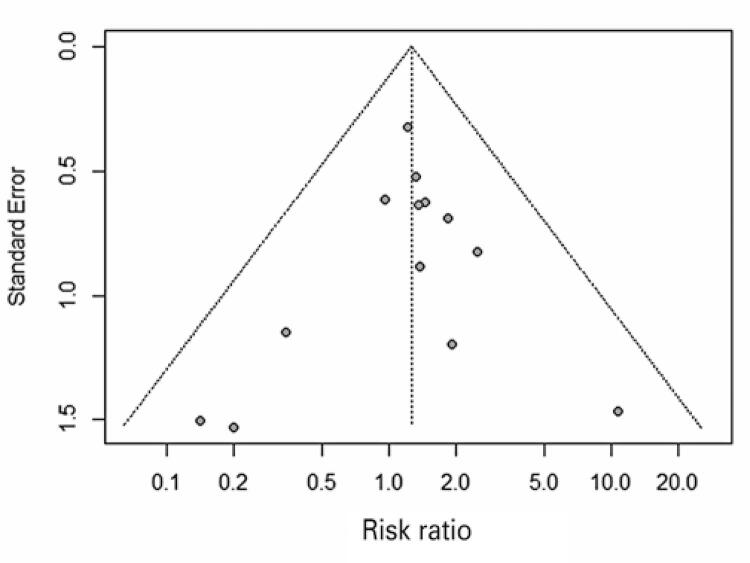
Funnel plot of outcome symptomatic intracranial hemorrhage

In the subgroup analysis, patients were stratified as to the time at which the outcome was assessed. No significant difference was observed between the intervention and the comparison in the 24-36 hour subgroup (3.71% *versus* 3.26%; RR of 1.17; 95%CI: 0.65-2.09; I ^2^ =0%; p=0.47; ten studies; 1,463 patients), and in the 90 days subgroup (6.05% *versus* 4.60%; RR of 1.34; 95%CI: 0.83-2.15; I ^2^ =0%; p=0.88; four studies; 1,242 patients). Furthermore, no difference was observed between subgroups. Heterogeneity was null for the subgroups. No statistically significant publication bias was observed (p=0.6312) ( Figure 7 ).

#### Revascularization

**Figure 8:**
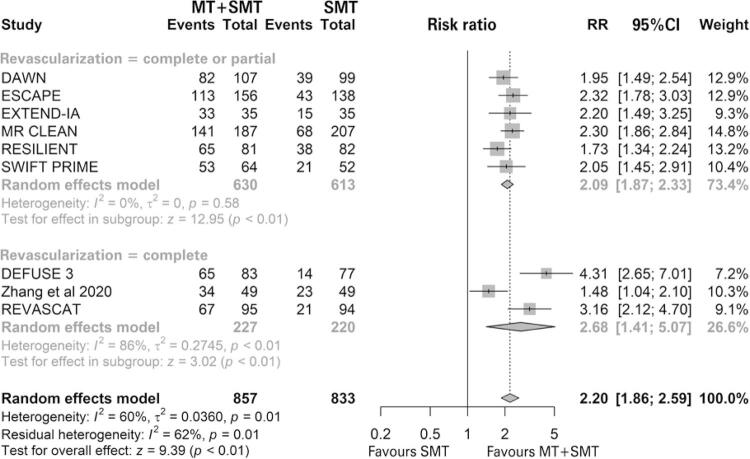
Forest plot of the meta-analysis of the revascularization outcome, stratifying studies by complete and complete or partial revascularization

MT: mechanical thrombectomy; SMT: standard medical treatment; RR: relative risk; 95%CI: 95% confidence interval.

Nine studies were included in the revascularization analysis, and all of them favored MT associated with standard medical treatment with statistical significance. The study by Zhang et al., ^( 57 )^ was evaluated only for this outcome and it was the only study that did not report the time that this datum was collected. Seven studies evaluated this outcomes at 24 hours and one at 27 hours. ^( 31 )^ In the meta-analysis, MT associated with standard medical treatment significantly increased the risk of patients having revascularization compared with standard medical treatment only (76.2% *versus* 33.85%; RR of 2.20; 95%CI: 1.86-2.59; p<0.01; I ^2^ =60%; p=0.01; nine studies; 1,690 patients) ( Figure 8 ).

**Figure 9:**
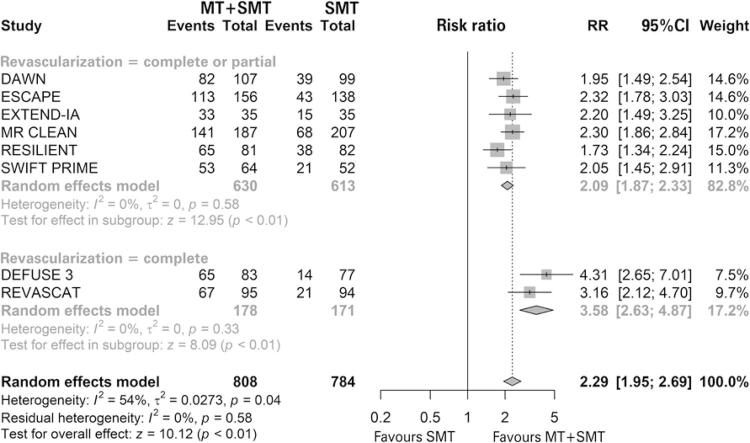
Forest plot of the meta-analysis of the revascularization outcome without the study, (57) stratifying studies by complete and complete or partial revascularization

MT: mechanical thrombectomy; SMT: standard medical treatment; RR: relative risk; 95%CI: 95% confidence interval.

In the subgroup analysis, patients were stratified into complete and complete or partial revascularization. We observed a difference between the intervention and the comparison in the complete or partial subgroup (77.30% *versus* 36.54%; RR of 2.09; 95%CI: 1.87-2.33; p<0.01; I ^2^ =0%; p=0.58; six studies; 1. 243 patients), and in the complete subgroup (73.13% *versus* 26.36%; RR of 2.68; 95%CI: 1.41-5.07; p<0.01; I ^2^ =86%; p<0.01; three studies; 447 patients). Furthermore, no difference was observed between the subgroups as the confidence intervals were compared. Due to the high heterogeneity, a sensitivity analysis was performed by excluding the study, ^( 57 )^ which did not report the time that this datum was collected. In this analysis, there is a difference between the intervention and the comparison in the complete or partial subgroup (77.30% *versus* 36.54%; RR of 2.09; 95%CI: 1.87-2.33; p<0.01; I ^2^ =0%; p=0.58; six studies; 1,243 patients) and in the complete subgroup (74.16% *versus* 20.47%; RR of 3.58; 95%CI: 2.63-4.87; p<0.01; I ^2^ =0%; p=0.33; two studies; 349 patients) ( Figure 9 ). The difference was significant between the subgroups and indicated that revascularization status may act as an effect modifier.

#### Quality assessment

In the methodological quality assessment of the studies (Appendices H to L), the overall risk of bias was assessed for all outcomes in most of the included studies in the SC or HRoB categories. This was due to domains 1 and 2 of the RoB 2.0 tool, which address the randomization process and deviations from intended interventions, respectively. ^( 48 - 57 , 60 , 61 )^ However, for some studies, for the outcomes functional independence, symptomatic intracranial hemorrhage and revascularization, the assessments categorized with SC or HRoB were also influenced by domain 4 of the outcome measure (Appendices J to L). ^( 48 , 50 , 53 , 58 )^ Only one study presented assessments of SC and HRoB in all domains for the outcomes all-cause mortality and symptomatic intracranial hemorrhage, because it was available only in summary form, and not all information needed for the assessment was presented. ^( 56 )^ Three studies had the overall risk of bias assessed with the LRoB category for all outcomes. ^( 46 , 47 , 59 )^

The trial with the most patients randomized, MR CLEAN, was assessed with some concerns on all outcomes for overall risk of bias, because the trial was reported to have slightly unbalanced randomization resulting in more patients in the control group. ^( 60 )^

The quality of the evidence was considered low to moderate (Appendix M). All outcomes were downgraded -1 for risk of bias, and the outcome symptomatic intracranial hemorrhage was the only one to be downgraded -1 for imprecision, because it is noted that the magnitude of effect was based on a small number of events.

## DISCUSSION

In this study, mechanical thrombectomy associated with standard medical treatment compared with standard medical treatment only for patients with acute ischemic stroke resulted in a lower risk of death (16.81% *versus* 20.13%; RR of 0.85; 95%CI: 0.72-0.99; p=0.04), higher risk of patients being functionally independent after 90 days of treatment (45.65% *versus* 27.45%; RR of 1.65; 95%CI: 1.41-1.91; p<0.01), and higher risk of revascularization (76.2% *versus* 33.85%; RR of 2.20; 95%CI: 1.86-2.59; p<0.01). The outcome symptomatic intracranial hemorrhage showed no statistically significant difference (4.78% *versus* 3.88%; RR of 1.27; 95%CI: 0.88-1.83; p=0.21). The results found in this study are important to support decisions about alternative that are more appropriate for clinical practice, since the use of the intervention that was favorable in terms of the final outcomes. Moreover, in this study, as recanalization has already been associated with a good clinical outcome, the intervention favoring partial or complete revascularization also contributes to the evidence of better clinical outcome. ^( 62 )^ However, one should be cautious with these results regarding patients with vertebrobasilar artery occlusion, as further studies are needed in this population to prove the safety and efficacy of this treatment, as the only study that evaluated this population did not show good results. ^( 51 )^

In two other systematic reviews that compared medical treatment and endovascular therapy, including first and second-generation devices, eight randomized clinical trials were evaluated. These reviews showed that patients who were treated with endovascular therapy had better functional independence within 90 days. However, they showed no statistically significant difference regarding all-cause mortality and the outcome symptomatic intracranial hemorrhage. ^( 24 , 25 )^ Furthermore, endovascular thrombectomy was associated with significantly higher rates of angiographic revascularization within 24 hours. ^( 25 )^ These results were similar to those found in this review, differing only regarding to all-cause mortality.

In another recent systematic review, which evaluated the effect of MT in acute ischemic stroke patients with large vessel occlusion, 11 randomized clinical trials were evaluated. From the meta-analysis of these studies, it was demonstrated that the association of MT, and improved medical treatment leads to a statistically significant reduction in 3-month mortality (RR of 0.83; 95%CI: 0.69-0.99; p=0.04).This was a result that is similar to that of this study. ^( 63 )^ However, unlike this systematic review, ^( 63 )^ this study did not restrict the population to patients with large vessel occlusion, as it sought to evaluate the efficacy and safety of the intervention for all patients with acute IS. Moreover, the present literature search was performed in more databases, such as Embase, a factor that also contributed to the inclusion of more studies in the analysis.

The MT has already been approved a few years ago for the treatment of acute IS in countries such as United States, Canada, and Brazil. ^( 16 , 64 )^ However, the decision for incorporation into the public health system in Brazil occurred this year. ^( 65 )^ The Brazilian RESILIENT study, included in this article, contributed to this decision by showing favorable results to the technology, despite the limitations of a developing country. Further studies in developing countries may be needed to support the decision to incorporate this intervention, since most studies were conducted in developed countries. Moreover, in addition to these studies, investment should be made in economic studies, since this technology has higher costs. ^( 19 )^ In a cost-utility analysis in Canada, it was found that IT with MT is cost-effective when compared with IT only in patients who had large-artery IS. The incremental cost-effectiveness ratio was C$ 11,990 per quality-adjusted life-year over 5 years. ^( 66 )^

Similar to other systematic reviews, this study had limitations in outcome analysis due to differences in design, methodology, and clinical and neuroimaging inclusion criteria across studies, as noted in the general characteristics of the included studies. In this study, we evaluated the main outcomes (mortality and functional independence) in relation to eligibility for IT, since some studies were only conducted in eligible patients. Therefore, it is also important to evaluate the outcomes in relation to some other variables, such as time since onset of symptoms and treatment with thrombectomy, to be able to assess the effect of the intervention when performed in the short and long term. These factors are important in defining the recommendation criteria for the technology.

## CONCLUSION

Mechanical thrombectomy, combined with standard medical treatment, seem to be safe and effective for the treatment of patients with acute ischemic stroke when compared with standard medical treatment only.
